# Genome Sequence of *Prosthecochloris* sp. Strain ZM and *Prosthecochloris* sp. Strain ZM-2, Isolated from an Arctic Meromictic Lake

**DOI:** 10.1128/MRA.01415-18

**Published:** 2018-11-29

**Authors:** Denis S. Grouzdev, Vasil A. Gaisin, Maria S. Krutkina, Irina A. Bryantseva, Olga N. Lunina, Alexander S. Savvichev, Vladimir M. Gorlenko

**Affiliations:** a Research Center of Biotechnology, Russian Academy of Sciences, Moscow, Russia; Queens College

## Abstract

Draft genome sequences of green-colored and brown-colored green sulfur bacteria (GSB), *Prosthecochloris* sp. ZM and *Prosthecochloris* sp. ZM-2, respectively, which were isolated from the Arctic meromictic lake Zeleny Mys, were sequenced. The genomes' differing gene compositions determine the differences in the bacteriochlorophyllic compositions of these bacteria.

## Abstract (precis)

Draft genome sequences of green-colored and brown-colored green sulfur bacteria (GSB), *Prosthecochloris* sp. ZM and *Prosthecochloris* sp.

## ANNOUNCEMENT

The particular pigment compositions of green-colored and brown-colored green sulfur bacteria (GSB) allow them to occupy different photic niches (1). Here, we announce the draft genome sequences of both brown and green GSB, *Prosthecochloris* sp. ZM and *Prosthecochloris* sp. ZM-2, respectively. These bacteria were isolated from the Arctic meromictic lake Zeleny Mys, known as “Green Cape,” which is periodically supplied by marine water through a channel from the Kandalaksha Gulf (66.53031, 33.09498). *Prosthecochloris* sp. ZM was isolated from the chemocline of the lake and had bacteriochlorophyll *e* (713 nm) as the main photosynthetic pigment. *Prosthecochloris* sp. ZM-2 was isolated from a microbial mat in the coastal zone of the lake and had bacteriochlorophyll *d* (730 nm) as the main photosynthetic pigment. The wavelength of the pigments was determined in 50% glycerol cell suspension using a spectrophotometer (SF-56A, OKB Spectr). Both strains were isolated and maintained under 25 to 35°C in light (2,000 lx) using the medium (g liter^−1^) 0.5 NH_4_Cl, 0.5 KH_2_PO_4_, 0.2 MgCl_2_ · 6H_2_O, 0.1 CaCl_2_ · 2H_2_O, 20.0 NaCl, 0.3 KCl, 3.0 NaHCO_3_, 1.0 Na_2_S_2_O_3_ · 6H_2_O, 0.7 Na_2_S · 9H_2_O, 0.1 yeast extract, and 0.5 sodium acetate and Pfennig’s vitamin solution and trace-element solution, 1 ml each.

Genomic DNA was extracted using Wilson’s (2) method with minor modifications. Briefly, the cell pellet was resuspended in 400 μl of Tris-EDTA (TE) buffer; then 25 μl of 10% SDS and 20 μl of proteinase K (20 mg/ml) solution were added, and the mixture was incubated at 37°C for 60 min. After incubation, 125 μl of 4 M NaCl, 160 μl of 5% cetyltrimethylammonium bromide (CTAB), and 20 μl of RNase (10 mg/ml) were added. The mixture was then incubated for 10 min at 65°C and cooled to room temperature; then the mixture was treated with chloroform followed by centrifugation for 10 min at 9,000 × *g*. DNA from the supernatant was recovered by adding 0.6 volume of isopropanol. The dried DNA was dissolved in 50 μl of deionized water. Libraries were constructed with the NEBNext DNA library prep reagent set for Illumina following the kit’s protocol. Sequencing was undertaken using the Illumina HiSeq 1500 platform with single-end 230-bp reads. A total of 10,285,213 and 10,102,311 reads were obtained from *Prosthecochloris* sp. ZM and *Prosthecochloris* sp. ZM-2, respectively. Raw reads were quality checked with FastQC v 0.11.7 (http://www.bioinformatics.babraham.ac.uk/projects/fastqc/), and low-quality reads were trimmed using Trimmomatic v 0.36 (3). The quality-filtered reads were *de novo* assembled with SPAdes v 3.11.0 using the default settings (4), which yielded 40 and 160 contigs larger than 500 bp for strains ZM and ZM-2, respectively. The multidraft-based scaffolder MeDuSa (5) was used to generate scaffolds from the contigs and to perform the mapping against Prosthecochloris aestuarii DSM 271^T^ (ENA accession no. GCA_000020625) and *Prosthecochloris* sp. HL-130-GSB (ENA accession no. GCA_002113825) as reference genomes for strains ZM and ZM-2, respectively. The final assembled 2,661,000-bp-long genome comprised 7 scaffolds with an *N*_50_ value of 2,653,521 bp, an average coverage of 320×, and a GC content of 49.9% for the strain *Prosthecochloris* sp. ZM. The final 2,403,542-bp-long genome comprised 71 scaffolds with an *N*_50_ value of 2,141,884 bp, an average coverage of 390×, and a GC content of 55.5% for the strain *Prosthecochloris* sp. ZM-2. Annotations of the scaffolds were carried out using the NCBI Prokaryotic Genome Annotation Pipeline (6), which identified 2,529 genes, 2,392 coding sequences, 85 pseudogenes, and 46 tRNA genes for the strain ZM and 2,258 genes, 2,146 coding sequences, 60 pseudogenes, and 46 tRNA genes for the strain ZM-2.

**FIG 1:**
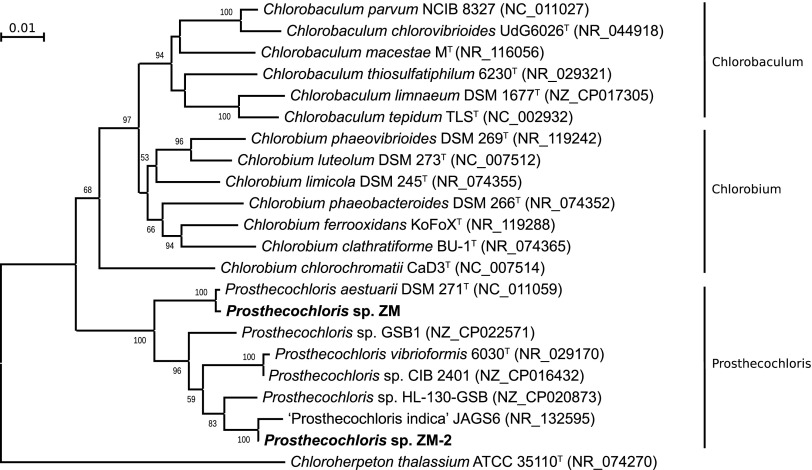
Neighbor-joining phylogenetic tree inferred from 16S rRNA gene sequences showing the position of both strains, *Prosthecochloris* sp. ZM and *Prosthecochloris* sp. ZM-2. The total 1,383 positions were analyzed. Bootstrap values are based on 1,000 replicates.

*Prosthecochloris* sp. ZM is a brown-colored strain which was identified as species P. phaeoasteroidea according to morphological properties (7). However, the strain ZM belongs to the species P. aestuarii according to a 16S rRNA phylogenetic tree (Fig. 1), which was reconstructed by a neighbor-joining method (8) using MEGA7 (9). Additionally, the strain has an 87.7% value of DNA-DNA hybridization with P. aestuarii DSM 271^T^, which was calculated using an *in silico* method (10). The genome of the strain ZM contains the gene *bciD*, which encodes an enzyme that is involved in the synthesis of bacteriochlorophyll *e*, unlike P. aestuarii DSM 271^T^. *Prosthecochloris* sp. ZM-2 belongs to the clade “*Prosthecochloris indica*” JAGS6 (Fig. 1). Both announced genomes lack genes of the *sox* system for thiosulfate oxidation, but they contain the gene of sulfide:quinone oxidoreductase for sulfide oxidation. Additionally, they contain *nif* genes for the nitrogenase complex. Genes for biosynthesis of gas vesicles were absent from both genomes.

### Data availability.

This whole-genome shotgun project has been deposited in DDBJ/ENA/GenBank under the accession no. PDNX00000000 for *Prosthecochloris* sp. ZM and PDNY00000000 for *Prosthecochloris* sp. ZM-2. The raw FASTQ reads have been deposited in the NCBI SRA database under the accession no. SRR8090338 for *Prosthecochloris* sp. ZM and SRR8090339 for *Prosthecochloris* sp. ZM-2.
